# Efficacy of Telemedicine Utilization for Cardiac Outpatients' Care during the Pandemic of COVID-19: A Large Center Experience in the Wave of the Pandemic

**DOI:** 10.1155/2022/4156436

**Published:** 2022-05-17

**Authors:** Wesam A. Alhejily

**Affiliations:** ^1^Departments of Medicine (Division of Cardiology) Faculty of Medicine, King Abdulaziz University Hospital, Jeddah, Saudi Arabia; ^2^Cardiology Division at Dr Sulaiman Alhabib Medical Group, Saudi Arabia

## Abstract

**Introduction:**

Synchronous telemedicine using teleconferencing may play a role in clinical care. In a tertiary care center, video conferencing-enhanced virtual clinics were established via a new application platform. They were introduced during COVID-19 pandemic to connect patients to trained health-care providers via a secured line. While maintaining patients' privacy, they were theorized to offer effective communications and continuous clinical care. In this cross-sectional cohort study, we ought to examine the impact of service and the assistance they may offer to cardiac patients in the outpatient setup.

**Methods:**

A prospective cohort study looking at all video-conferencing virtual clinics' visits during the pandemic with primary focus on cardiac outpatient clinic, addressing primary endpoints of need for admission or emergency visits from cardiac demises during the pandemic and secondary endpoint of patient satisfaction based on patients' experiences.

**Results:**

A total of 6000 live care video-based chats were made over 10 months period from March 10^th^, 2020, to January 30^th^, 2021, among which 277 patients were evaluated in the virtual cardiac clinic, of these 193 (69.7%) were males, with mean age of 48 ± 15.60 (22.3%), patients were requested to present to clinic for further evaluation and testing, 20 (7.2%) patients were asked to visit the emergency room, of whom 8 (2.8%) patients were hospitalized. All 8 were admitted for high-risk findings that require immediate medical attention, 4/8 underwent cardiac catheterization, mean duration of admission was 2 ± 1 days. When compared to regular walk-in care, there was a statistical difference in admission rate and emergency visits *p* = 0.001 and *p* = 0.0001, respectively, both were statistically higher in the virtual clinic. The satisfaction rate in a 5-scale grading system was 97% of 4 and above recommending the continuation of the service beyond pandemic time.

**Conclusions:**

Video-enhanced virtual cardiology clinic works safely and efficiently during COVID-19 pandemic with a difference in admission rate and ER visits when compared to walk-in clinic. It can be used during nonpandemic time to reduce load on hospital and clinic crowdedness. It also decreases the chances of COVID-19 transmission indirectly by reenforcing physical distance.

## 1. Background

“Telemedicine” means diagnosing and treating patients with the help of information technology and distance communication systems [1]. This involves any kind of medicine practiced at a distance, with the help of any means of communication such as telephone, fax, and electronic mail (email) technology, as well as the use of interactive live streaming videos and audios. It helps doctors or other health-care providers communicate with their patients at their locations without the need to show up in person to hospitals. It has the gramine of monitoring, diagnosing patients at home, treating, and counseling. This eventually leads to cost-effectiveness by saving money and time for end users [2, 3].

In the field of cardiology, electrocardiograms were transmitted over ordinary analogue telephone lines in 1940s [4] with medical advice service for sea craft and voice radio. Although the term “Telemedicine” was coined back to 1977, its real time usage was not tangible before mid-90s. 2-way closed-circuit television systems were used in the 1960s to transmit radiographs and evaluate patients. These nowadays have been replaced by low-cost, personal computer–based, and smartphone solutions for videoconferencing [5]. Transmission of physiological data from clinics or patients' homes or from inaccessible sites in geographically remote regions became visible, and it is used to treat patient with acute emergencies like stroke with specific time-sensitive approach [6, 7]. The World Health Organization has established many programs globally that are based on Telemedicine to provide services in remote areas of far countries [8–12].

In the wakeup of COVID-19 pandemic, social distancing became essential part of preventing the spread of the raging viral illness, making “Telemedicine” more needed than ever before [13]. Many health-care centers have adopted many solutions to avoid hospital overflow and to open more space for infected patients. That included cutting down the numbers of outpatient's visits and elective procedures. Virtual clinics were established as alternative solutions to cover the demand needed for outpatients' care [14–20].

In our center, a new service called “LiveCare” was added to hospital online application, where patients can book appointments in virtual clinics to attend with any specialty including primary care. Patient can choose between video, voice conferencing, or type-in chatting, regular phone calls were also permitted and scheduled throughout the regular time of any clinics. From a technical point of view, LiveCare was established on Habib Medical Group website (HMG) early March 2020.

**Figure 1:**
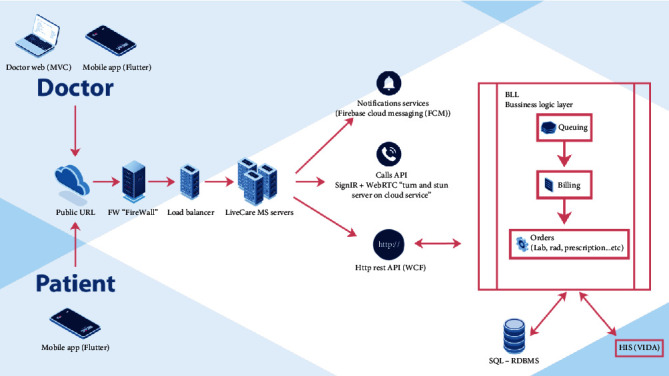
Hardware and software used to integrate live care to health information system. MVC = model view controller; URL = uniform source locator; MS = Microsoft servers; API = application program interface; HTTP = hypertext transfer protocol; SQL = structured query language; RDBMs = relational database management systems; HIS = health information system.

HMG application is accessible on smartphones and can be found on Apple® and Android® applications' stores and is connected to VIDA, the health information system of HMG hospitals, structurally, the system has hardware and software backbones which are designed as shown in Figure 1.

**Figure 2:**
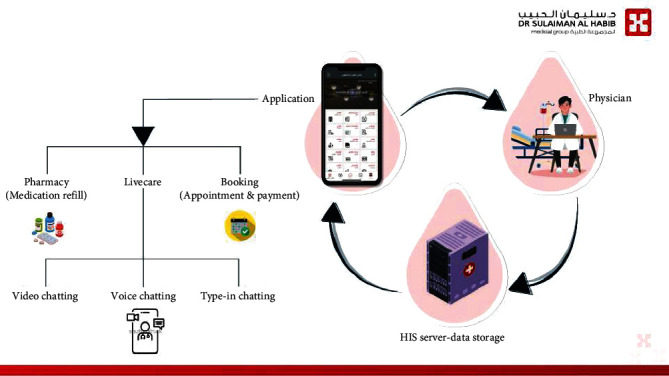
Habib Medical Group (HMG) application integrated to Vida (HIS) and its core functionality, which enables direct communication between patients (who has the application) and hospital services.

Live care is a flutter-based mobile application utilizing model view controller, the last describes the separation of the app's data (Model) from the app's interface (View) from the app's logic (Controller). Patients and doctors are connected via cloud-based “Uniform Source Locator” that connects to Microsoft-based servers. Servers provide three main functions, application program interface calls, notification services, and windows-based communication foundation, the last connects to business logic layer, all connected to the HIS (Vida).  Figure 2 shows the integration of HMG application that has LiveCare (the telemedicine services) to health information system (HIS).

**Figure 3:**
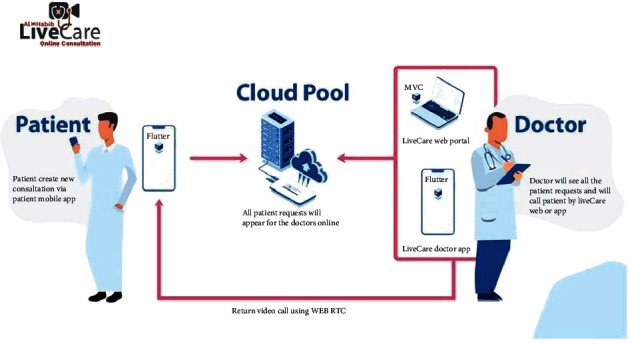
LiveCare (Telemedicine) platform. MVC = model view controller; RTC = real-time communication.

Functionally, the telemedicine (LiveCare) system builds on three platforms (Figure 3):

1. Patient Application: used by the patients to login to his\her medical file, patients can select LiveCare Service, choose the clinic, pay the consultation fees, and place the consultation request in the queue. Patient Application is fully integrated with the health information system (Vida) that allows the patient to view the medical file from the vital signs-up to the radiology images with r more than 100 other services
2. LiveCare Doctor Portal: used by doctors inside the hospital on the intranet to view the queue of the consultation requests according to the clinic and call the patient over the video call Module using the Web Real-Time Communication (Web RTC) technology. The LiveCare Portal is a web application built on Asp. Net model–view–controller (*MVC*) technology and fully integrated with the health information system, allowing doctors to view patient's complete medical history
3. Doctor Application: doctors outside the hospital use to view the queue of the consultation requests according to the clinic and call the patient over the video call Module using the Web RTC technology. The Doctor Application is a mobile application built on Flutter technology and fully integrated with the health information system, allowing the doctor to view the patient's complete medical history

Other platforms' applications were used in Saudi Arabia with several studies which were conducted to assess the impact of telemedicine on patient care during COVID-19 pandemic; among these, one used a questionnaire to health-care providers, showing that most agreed that “Telemedicine can reduce unnecessary outpatients' visits, depending on the specialty addressed” and “can provide care for most chronic patients” but still has barrier such as legality, diagnostic concordance, and technical issues [21]. In “Taif”, a small city with 700 thousand population, diabetic care was maintained during the pandemic using telemedicine and it was thought that the care has positive impacts on patients' blood sugar control [22]. However, in a meta-analysis looking at more than 43 studies of using Telemedicine in the Middle East, it was found that progress made in the utilization of telemedicine was insufficient and varies across countries. Barriers related to organizational, cultural, financial, individual, technological, legal, and regulatory challenges were preventing this service from being fully used [23].

During the pandemic, Saudi health authorities have provided many telehealth applications using voice or video chatting conferences on smartphones to mitigate the spread of infection, these include mobile applications such as Sehhaty, Tawakkalna, Mawid, Tabaud, and Tetamman, all of which have been found effective tools to facilitate delivering healthcare to infected persons with COVID-19 and tracking of newly confirmed COVID-19 patients. Study has shown that it helped flatten the COVID-19 curve in Saudi Arabia [24].

In this study, we will shed light on our experience utilizing a video conferencing telecommunication platform as part of “liveCare” to establish cardiac outpatient services without the need of patients' actual attendance in a private tertiary center in Riyadh, the capital city of the Kingdom of Saudi Arabia.

## 2. Methods

Prospective cohort study looking at all patients visited the cardiology clinic virtually using video-assisted chatting compared to real-time walk-in clinics during COVID-19 pandemic, patient's demographic and clinical data were stored synchronously on a computer-based system used by the hospital. Study was ethically approved by Institutional Review Board (IRB) ethical committee, patients were consented to have their data anonymously collected from the Information Health System and from the online application “LiveCare.” All information was Health Insurance Portability and Accountability Act “HIPPA” complaint. Patients attending any clinic were scheduled to receive an SMS message within 24 hours from the time of their visits with a link to access a satisfaction scoring system. Range of satisfaction includes 5 points of the following: “1-Not at all Satisfied”, “2-Partly Satisfied”, “3-Satisfied”, “4-More than Satisfied,” and “5-Very Satisfied,” numbering 1 to 5 as an interval scale. The same scale was used to assess several points that pertain to the clinic, including physician performance like listening to patient complaints and addressing them appropriately, formulating a sound plan of care that is understandable, and establishing a professional rapport with patients. Accessibility and quality of other services like administration, scheduling appointment, diagnostic laboratory tests, imaging, and pharmacy were also evaluated. Finally, the application itself as a platform of telemedicine was addressed with two questions “weatherwas it easy and user friendly?” and secondly, “were there any technical issues related to connectivity or responsiveness?” Finally, overall rating to support continuation of the service was collected using the same scaling system. Clinical data were collected manually and anonymously in a spread sheet and reviewed monthly to reflect the data entered in both the health information system and LiveCare.

Video-conferencing virtual clinic compared to actual OPD visit clinical information was analyzed looking at the primary endpoint of need for admission from cardiac demises during the pandemic. Secondary endpoint of patient satisfaction based on patient experiences was obtained. Analysis of variance “ANOVA” was done using mean results, with power of 95%, a margin of error of 5% and assuming 1000 population, with 50% proportion, we estimated sample size using (*n*) which is calculated according to the formula: *n* = [*z*2∗*p*∗(1 − *p*)/*e*2]/[1 + (*z*2∗*p*∗(1 − *p*)/(*e*2∗*N*))], where *z* is equal to1.96 for a confidence level (*α*) of 95%, *p* is the proportion (expressed as a decimal), *N* is the population size, and *e* is the margin of error. *z* = 1.96, *p* = 0.5,*N* = 1000,*e* = 0.05, and *n* = [1.962∗0.5∗(1 − 0.5)/0.052]/[1 + (1.962∗0.5∗(1 − 0.5)/(0.052∗1000))] *n* = 384.16/1.3842 = 277.54. The sample size (with finite population correction) is equal to 277 of normally distributed data, IBM-SPSS® version 22 was used to compute all statistical analysis.

## 3. Results

Study was conducted from March 2020 to January 2021, in the span of 10 months a total of 6000 calls were received by LiveCare which was mounted on the main hospital application, a promo advertisement was added at the login of the application to introduce the service after the announcement of curfew at the peak of COVID-19 pandemic in mid-March 2020 in the Kingdom of Saudi Arabia.

## 4. Patients' Characteristics and Reason for Calls

More than 277 video-assisted virtual clinic visit cases were reviewed in the cardiology outpatient clinic using LiveCare platform, of these patients, 193 (69.7%) were males, with mean age of 48 ± 15. With regard to chief complaints, 10.5% presented with atypical chest pain, 7.2% presented with typical angina, and 2.2% were having main symptoms of dyspnea. 10.8% had a main chief complaint of palpitation. Uncontrolled hypertension was the main cause of the call in 12% of the times. Calls for medication refill were the dominant service provided, representing 32.5%. There were 12.6% calls related to counseling. Only 1.4% of calls were related to COVID-19 infection and its impact on cardiac health.  Table 1 summarizes the chief complaints of callers.

## 5. Cardiac Risk Factors among Callers

Risk factors among callers were diabetes in 16.2%, hypertension in 18.4%, and hyperlipidemia in 10.5% of the patients. History of prior percutaneous coronary interventions with stenting (PCI) was present in 7% and coronary artery bypass surgery was present in 2.5% of patients, respectively.  Table 2 illustrates the preexisting conditions of patients' population.

## 6. Main Medical Services Provided

Medical services provided encompassed medication refills in 49% of patients and investigations were ordered in 58% of patients, and there was an urgent ER visit request in 20 patients and 8 patients were admitted.  Table 3 shows a pie chart emphasizing the main services provided.

## 7. Results of Primary and Secondary Endpoints for Virtual vs. Real-Time Clinics and Patients' Satisfaction Rate

When compared to the regular walk-in clinic, there were more need for ER visits 5 vs. 20 for liveCare patients, and admission rate was doubled 4/277 vs. 8/277 patients, no deaths were noted in any group. Among the admitted to the hospital, four underwent cardiac catheterization with successful revascularization, and the other four were with decompensated heart failure with need for in-hospital care due to worsening renal function, or lack of response to regular heart failure medications particularly diuretics. Statistically, *p* = .0001 and *p* = .001 were significant for ER visits and admission, respectively. The satisfaction rate in a scale of 5 was 97% with score of 4 and above requesting the continuation of this services, application was user friendly and easy to execute for more than 93% of end users with a rate of 3 and above, indicating positive experience from patient's standpoint.  Table 4 elaborates the results of primary and secondary endpoints.  Table 5 summarizes the overall satisfaction rate among different parameters that are related to patients' experiences.

## 8. Discussions

Face-to-face telemedicine is not new, it was used before in psychotherapy- and psychiatry-related services, also in remote underserviced communities [25–30]. Despite being evaluated before, this is the first trial of its kind to address the effectiveness of telecardiology clinic utilizing video-assisted chatting as mean way of communication, the service scope was vast covering all aspects of cardiovascular medicine practice. 277 patients were evaluated effectively, of which 20 (7.2%) patients needed to come to the ER for concerning symptoms, of these eight patients (2.89%) needed urgent admission to the hospital. The rate of admission was statistically significant compared to the admission rate from the regular walk-in clinic. The rate of satisfaction was exceedingly high, we believe that patients valued the surface provided to them via liveCare platform as more than 97% were with continuation of the service.

Comparing virtual telecardiology services to regular outpatient services, there was lower numbers of patients physically present in the clinics, and time consumed in clinic was shorter with more efficiency in serving more patients. It also helped reduced patient-to-patient contacts in the waiting area which thought to be necessary to reduce COVID-19 transmission, this also was more convenient to patients avoiding a trip to hospital. It also encourages more patient to visit the clinics given the easy accessibility to their primary physicians and other health-care providers. The cons include lack of face-to-face communication, inability to perform direct physical examination, and hemodynamic assessment, in addition to uncertainty related to patients' clinical diagnoses.

**Figure 4:**
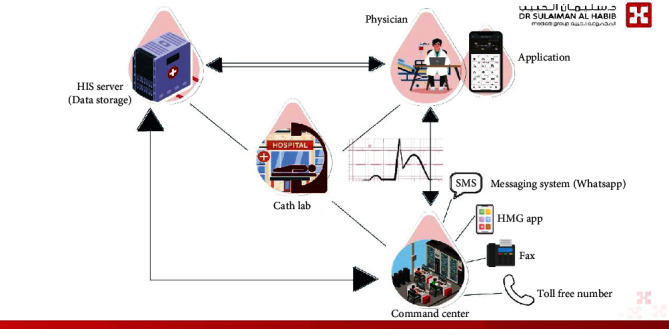
STEMI telecardiology care.

In addition to the virtual outpatient clinics and online automated services, and due to need for acute cardiac care related to acute coronary syndrome, like ST elevation myocardial infarction (STEMI), we have introduced the “STEMI Tele-Cardiology”, in cases where patients' symptoms are suspected to be due to heart attack, patient can get to this service by calling a toll-free number or activate the urgent care services using the hospital application. In addition to that, a trained team in the command center can accept calls/fax/SMS messages from any nearby hospital to help serving for acute cardiac care. Once the service is activated, an ambulance will be dispatched to the location of the patient using the nearest possible route on the map screen in the command center after defining caller location, and an ECG is done as soon as possible before it gets sent wirelessly to the on-call cardiologist, this can be sent to hospital application dedicated for clinicians to access its services to be sure that data are safely stored, the cardiologist can then activate the catheterization lab ahead of time to be ready to receive the patient (Figure 4). In a recently published study, the service was valuable in reducing ischemia time in patient with STEMI [31].

Applying telecardiology during the COVID-19 pandemic helped the continuation of cardiac care to a rather vulnerable population of patients and has proven to be effective in a couple of previously published trial and systematic review [32, 33], telecardiology albeit did not improve mortality, it was able to reduce emergency visits and reduce need for rehospitalization particularly in heart failure patients, this study attests to the high need of such clinics even during normal life given how convenient it will be for patients to meet their cardiologist without the need to be physically available in the clinic. The rate of admission via the clinic was higher than expected and that could be related to the patient presentations and clinical symptoms in addition to lack of hands-on assessment by clinicians, the last makes providers more concerned that they might have missed something serious, these factors led to more ER visits and subsequently more admissions. Telecardiology clinic despite its inherited limitations related to limited access to critical information like vital signs, clinical exam findings, and closer eye assessment was successful in this short series to treat patients effectively and efficiently. It is also reenforced social distance as mean of reducing COVID-19 transmission from person to person. Finally, no death reported among callers for the entire period of the study. It is worth mentioning that other modalities of telecardiology were used such as direct phone calls, type-in chatting, and automated services (medications refill), when compared to video chatting, they are cheaper and not technically demanding and to some patients more convenient.

There are quite some limitations to this study as the number of patients is relatively small, the area where the study was conducted in the City of Riyadh is considered one of the high socioeconomic regions. And affordability to the service was attainable by most known patients to the hospital. There are also some challenges to the service, including legality related to missing major findings that only will be picked up in hospital, there is also a socioeconmic disparity in acceptance and affordability related to financial constraints but also can be self-preferences. Prior experience in Telemedicine has highlighted these challenges very well addressing the need for unified approach that provides high-quality care with reduced cost and more accessibilities to public [34–43]. Larger study with more international centers could illustrate better the impact of diverse cultures and socioeconomic stratum on the provision of clinical care using video-assisted chatting as a mean of telemedicine in any clinical field.

## 9. Conclusions

In this prospective cohort study of video-assisted telecardiology clinic, the virtual clinic was successful in triaging cardiac patients and providing the services needed, based on their clinical presentations. There was excellent satisfaction rate among caller in more than 97% of callers. The rate of urgent ER visits and admission to the hospital during the curfew of COVID-19 was higher and was statistically significant compared to walk-in clinic with no increase in death rate.

## Figures and Tables

**Table 1:** Cardiac symptoms among callers to Virtual Cardiology clinic.

Symptoms	*N*	%
Atypical chest pains	29	10.5
Chest pain	20	7.2
Dyspnea	6	2.2
Palpitations	30	10.8
Syncope, dizziness	3	1.1
Uncontrolled HTN	33	11.9
Medication's refill	90	32.5
Volume overload	4	1.4
Labs results	21	7.6
Counselling	35	12.6
Wrong call	1	0.4
COVID-related	4	1.4
Sick leave	1	0.4
Total	277	100.0

**Table 2:** Cardiac risk factors and preexisting conditions.

Risk factor and preexisting conditions∗	*N*	%
Non	51	18.4
Dm	45	16.2
HTN	51	18.4
HLP	29	10.5
IHD	9	3.2
HF	5	1.8
Afib	11	4
Stroke	6	2.2
PAD	24	8.7
PCI	7	2.5
CABG	4	1.4
RHD	9	3.2
Lung disease	4	1.4
DVT/PE	17	6.1
Smoker	3	1.1
VHD_Mechanical valve	1	0.4
Total	277	100

^∗^Legends: DM: diabetes mellitus; HTN: hypertension; HLP: hyperlipidemia; IHD: ischemic heart disease; HF: heart failure; Afib: atrial fibrillation; PAD: peripheral arterial disease; PCI: percutaneous cardiac intervention; CABG: coronary artery bypass surgery; RHD: rheumatic heart diseases; DVT/PE: deep vein thrombosis/pulmonary embolism; VHD_Mechanical valve: valvular heart disease including mechanical valve prosthesis.

**Table 3:** Services provided to callers.

Services provided	*N*	
Medication refill	101	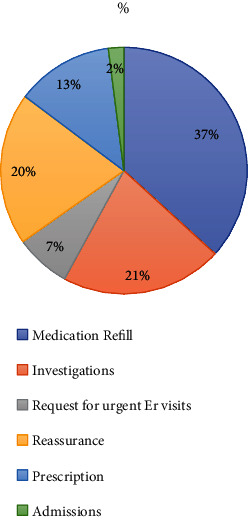
Investigations	58	
Request for urgent ER visits	20	
Reassurance	55	
Prescription	35	
Admissions	8	
Total	277	

**Table 4:** Admission and need for ER visits in virtual vs. regular clinics.

Clinic type	Regular walk-in clinic	Virtual clinic	*p* value
Admission	4/277	8/277	0.001
Need for ER visit	5/277	20/277	0.0001

**Table 5:** Satisfaction rate among different parameters of video-assisted cardiac virtual clinic in 277 cardiac patients.

Parameter(s)	(1) Not satisfied	(2) Partly satisfied	(3) Satisfied	(4) More than satisfied	(5) Very satisfied	*N*
Doctor was listening properly	2	20	48	117	90	277
Doctor provided a clear plan for medical care	4	25	48	111	89	277
Administrative issue related to file and appointments were executed properly	8	40	100	90	39	277
Diagnostics and pharmacy services were carried out smoothly	18	19	78	83	79	277
Application was user friendly	7	12	86	82	90	277
Difficulties and technical issues were solved while online	15	18	98	75	71	277
What is your overall satisfaction rate to have this service continued?	3	1	3	147	123	277

## Data Availability

The data are attached to the manuscript.
